# Microbeam methodologies as powerful tools in manganese hyperaccumulation research: present status and future directions

**DOI:** 10.3389/fpls.2013.00319

**Published:** 2013-08-20

**Authors:** Denise R. Fernando, Alan Marshall, Alan J. M. Baker, Takafumi Mizuno

**Affiliations:** ^1^Department of Botany, La Trobe UniversityBundoora, VIC, Australia; ^2^Analytical Electron Microscopy Facility, La Trobe UniversityBundoora, VIC, Australia; ^3^School of Botany, The University of MelbourneParkville, VIC, Australia; ^4^Graduate School of Bioresources, Mie UniversityMie, Japan

**Keywords:** Mn hyperaccumulator, microbeam analysis, Gossia, *Maytenus cunninghamii*, *Alyxia rubricaulis*

## Abstract

Microbeam studies over the past decade have garnered unique insight into manganese (Mn) homeostasis in plant species that hyperaccumulate this essential mineral micronutrient. Electron- and/or proton-probe methodologies employed to examine tissue elemental distributions have proven highly effective in illuminating excess foliar Mn disposal strategies, some apparently unique to Mn hyperaccumulating plants. When applied to samples prepared with minimal artefacts, these are powerful tools for extracting true ‘snapshot’ data of living systems. For a range of reasons, Mn hyperaccumulation is particularly suited to *in vivo* interrogation by this approach. Whilst microbeam investigation of metallophytes is well documented, certain methods originally intended for non-biological samples are now widely applied in biology. This review examines current knowledge about Mn hyperaccumulators with reference to microbeam methodologies, and discusses implications for future research into metal transporters.

## Introduction

The intrinsic value of plant hyperaccumulation as a resource for expanding fundamental knowledge is as equally well recognized as are its potential practical benefits (Brooks, 1987; Baker and Brooks, 1989; Baker et al., 1992; Brooks, 1998; Brooks and Robinson, 2000; Chaney et al., 2000; Pollard, 2000; Reeves and Baker, 2000; Lombi et al., 2001; Macnair, 2003; Whiting et al., 2004; Meharg, 2005; Reeves, 2005; Chaney et al., 2007). On a global scale, it is clear that natural systems are increasingly under threat from human activities, with dire predictions for the future survival of many biota. There is thus a pressing need to address habitat conservation of these extraordinary plants and to gain a better understanding of their ecology and evolutionary histories (Pollard, 2000; Reeves and Baker, 2000; Baker and Whiting, 2002; Boyd, 2004; Whiting et al., 2004). Macnair (2003) points out that notwithstanding the potential commercial benefit of hyperaccumulation yet to be realized in any real sense, “the phenomenon is of sufficient intrinsic interest that its investigation is justified without any immediate commercial applications.” Whilst economic and environmental factors are major drivers of scientific research in this area, it is likely that accessibility and ease of propagation also influence species selection for study. Ubiquitous herbaceous Ni hyperaccumulators in the Brassicaceae remain the most investigated subgroup to date (Brooks and Radford, 1978; Lee et al., 1978; Gambi et al., 1979; Baker, 1987; Baker and Brooks, 1989; Baker et al., 1992, 2000; Krämer et al., 1997; Brooks, 1998; Nicks and Chambers, 2000; Mengoni et al., 2003; Asemaneh et al., 2006).

The possible benefits of seeking to understand lesser-known hyperaccumulators with restricted distributions and/or more unusual hyperaccumulated elements include: (a) further illuminating hyperaccumulation *per se*, (b) obtaining novel perspective on the physiological roles of those hyperaccumulated elements essential to plant nutrition, (c) gaining new insights into plant metal specificity and detoxification. Around 22 Mn hyperaccumulators are known worldwide (Table 1), their highly restricted collective distribution centered almost entirely over New Caledonia and Eastern Australia. Reports of two herbaceous *Polygonum* species described as Mn-hyperaccumulating on Mn mine sites in China (Wang et al., 2007; Deng et al., 2010) are difficult to access and may require further confirmation. Among well confirmed Mn hyperaccumulators are seven trees and a shrub species from Australia (Bidwell et al., 2002; Fernando et al., 2009), seven woody plants from New Caledonia (Brooks, 1998), a tree and a herb native to China (Xue et al., 2004; Yang et al., 2008), a herb native to the USA (Min et al., 2007; Pollard et al., 2009) and a tree native to Japan (Mizuno et al., 2008). The single Malaysian tree species whilst documented has not been identified below generic level (Proctor et al., 1989). The identities of certain herbaceous Mn hyperaccumulators are yet to be verified taxonomically at the species level, while several trees within the Australian group are listed as threatened due to habitat loss. Given that conservation and research are underpinned by correct identification of hyperaccumulator species, plant taxonomy is integral to the topic. Currently it is an uncommon practice to provide herbarium vouchers on hyperaccumulator plants in published research; however, it should be encouraged in order to enable independent verification of species. The majority of Mn hyperaccumulators are woody plants with sclerophyllous leaves and xerophytic anatomies, traits strongly favorable to retaining leaf-tissue integrity for *in vivo* microprobe examination.

**Table 1:** **Current worldwide listing of Mn hyperaccumulators**.

**Plant (family) species (citation)**	**Native country**
(Araliaceae)	
*Chengiopanax* species formerly in *Eleutherococcus*	
^*^Chengiopanax sciadophylloides (1)	Japan
(Apocynaceae)	
*Alyxia rubricaulis* (2)	New Caledonia (NC)
(Celastraceae)	
*Denhamia* species formerly in *Maytenus*	
^*^Denhamia fournieri (2)	NC
*Denhamia cunninghamii* (3)	Australia (AU)
(Clusiaceae)	
^*^Garcinia amplexicaulis (2)	NC
(Myrtaceae)	
*Eugenia sp*.1 (2)	NC
*Eugenia sp.* 2 (4)	Malaysia
*Gossia* species formerly in *Austromyrtus*	
*Gossia bamagensis* (3)	AU
^*^G. bidwillii (5)	AU
*G. gonocla* (3)	AU
^*^G. fragrantissima (3)	AU
*G. lucida* (3)	AU
*G. sankowsiorum* (3)	AU
*G. shepherdii* (3)	AU
(Phytolaccaceae)	
^*^Phytolacca acinosa (6)	China (CH)
*P. Americana* (7)	USA
(Polygonaceae)	
*Polygonum pubescens* (8)	Eurasia (E)
*P. hydropiper* (9)	E/USA
(Proteaceae)	
*Beaupreopsis paniculata* (10)	NC
*Virotia* species formerly in *Macadamia*	
*Virotia angustifolia* (10)	NC
^*^V. neurophylla (10)	NC
(Theaceae)	
*Schima superba* (11)	CH

* Species examined by microprobe analysis.

References: 1, (Mizuno et al., 2008); 2, (Jaffré, 1977); 3, (Fernando et al., 2009); 4, (Proctor et al., 1989); 5, (Bidwell et al., 2002); 6, (Xue et al., 2004); 7, (Min et al., 2007; Pollard et al., 2009); 8, (Deng et al., 2010); 9, (Wang et al., 2007); 10, (Jaffré, 1979); 11, (Yang et al., 2008).

Plant Mn hyperaccumulation was originally defined by a notional threshold foliar Mn concentration of 10,000 μg g^−1^ dry weight (DW) (Baker et al., 2000). More recently, there has been argument supporting a downward revision (Baker et al., 2000; Fernando et al., 2009). It is widely accepted that Mn predominates in its lowest (+2) oxidation state *in planta*, and this has been confirmed for several Mn hyperaccumulators, using X-ray absorption near-edge spectroscopy (XANES) (Graham et al., 1988; Fernando et al., 2010). There are multiple physiological functions to which Mn is essential, primarily photosynthesis and oxidative stress mitigation. Based on crop plants, “normal” nutritional requirements for Mn are met at ~50–800 μg g^−1^ DW even though it is widely tolerated at concentrations above (Graham et al., 1988; Marschner, 2002; Foulds, 2003). This is in marked contrast to other trace metal nutrients. Manganese crop toxicity is a significant problem in certain regions of the world where soils are Mn-enriched and acidic (Heenan and Carter, 1977; Temple-Smith and Koen, 1982; Hung et al., 1987; Davis, 1996; González et al., 1998; StClair and Lynch, 2005). The ability of tolerant crop lines to sequester excess Mn, along with Mn sensitivity in other varieties is not well understood, nor is the observed variation in plant physiological responses. Therefore, Mn hyperaccumulators can be exploited to address these questions given they shine a novel light on Mn nutrition and could potentially yield important new information regarding specific Mn chelators and transporters.

The first reports of Mn hyperaccumulation occurred over 35 years ago upon discovery of the New Caledonian group (Jaffré and Latham, 1974; Jaffré, 1977, 1979). At that time, the now little-used term “hypermangansphores” was introduced to describe Mn hyperaccumulators. In the early 1980s a group of Japanese Mn-accumulating species was the subject of detailed physiological investigation, including microprobe analysis (Memon and Yatazawa, 1980, 1981, 1982, 1984). Over the past decade it has become apparent via *in vivo* microbeam studies at least that these are somewhat unusual among hyperaccumulators (Fernando et al., 2006a,b, 2008). Between 2002 and 2008, additional Mn hyperaccumulators were discovered in eastern Australia (Bidwell et al., 2002), China (Xue et al., 2004) and Japan (Mizuno et al., 2008), that has now led to recognition of several new species and renewed interest in the phenomenon. To date, the woody species have only been investigated through a series of ecophysiological studies, whereas the two herbaceous *Phytolacca* (Phytolaccaceae) species have mainly been examined in controlled experiments (Xue et al., 2005; Fernando et al., 2006b; Mizuno et al., 2006; Xu et al., 2006a,b, 2009; Fernando et al., 2007a,b). Unlike their herbaceous metal-hyperaccumulating counterparts, Mn-hyperaccumulating trees and shrubs are extremely slow growing and often difficult to propagate.

Pioneering studies on plant hyperaccumulation using widely accessible Ni-, Cd- and Zn-hyperaccumulating herbs provide an invaluable framework for current focus on Mn hyperaccumulation. This has led to: (a) the discovery of several new Mn hyperaccumulators, which has augmented the overall biogeographic knowledge base of hyperaccumulators by providing new perspective on Mn-accumulating taxa analogous to those with already well-established links to other metals (Baker and Brooks, 1989; Baker et al., 1992; Pollard et al., 2000, 2002; Fernando et al., 2009); (b) the discovery that foliar Mn detoxification varies specifically in a manner possibly unique to Mn hyperaccumulators (Fernando et al., 2008); (c) indication that the physiological mechanisms associated with excess Mn uptake and storage in herbaceous Mn hyperaccumulators are similar to those found in herbaceous hyperaccumulators of other metals (Küpper et al., 2000, 2001; Xu et al., 2006a,b); and (d) the pursuit to identify Mn-specific transporters, particularly in woody species where Mn is compartmentalized in highly localized vacuolar concentrations (T. Mizuno unpublished data).

## Metal localization methodologies applied to hyperaccumulators and other plants

Early studies utilizing microbeam techniques to investigate excess Mn sequestration in certain tolerant crop lines established that dermal tissues were primary deposition sites (Blamey et al., 1986; Edwards and Van Steveninck, 1987, 1988). Scanning electron microscopy energy dispersive spectroscopy (SEM/EDS), proton (or particle)-induced X-ray emission energy dispersive spectroscopy (PIXE/EDS) and synchrotron methodologies are by far the most widely used techniques for localizing excess metal deposition within plant tissues. To obtain reliable artifact-free data from *in vivo* methods such as these, it is imperative that sample preparations effectively immobilize cell metabolic and diffusional processes, thereby avoiding artifactual cell-content removal or relocation of diffusible elements (Marshall, 1980c; Morgan, 1980; Echlin, 1992; Marshall and Xu, 1998). This subject will be addressed in greater detail below. Other techniques including ^31^P-nuclear magnetic resonance (NMR) (Roby et al., 1988; Quiquampoix et al., 1993a,b) radioactive tracer studies (Lasat et al., 1996), and cell fractionation (González and Lynch, 1999) have also provided useful information regarding metal localization in plants. Mention should also be made of histochemical techniques utilized by Severne (1974) to locate foliar Ni *in situ* in a hyperaccumulator using dimethyl glyoxime stain; and by Horiguchi (1987) to localize oxidized Mn in rice plants by staining with benzidine.

*In vivo* microprobe investigation has been applied to hyperaccumulating plants to reveal that with the exception of Mn hyperaccumulators, primary sequestration occurs in non-photosynthetic tissues (Vázquez et al., 1992; Krämer et al., 1997; Küpper et al., 1999, 2000, 2001; Mesjasz-Przybylowicz et al., 2001; Lombi et al., 2002; Ager et al., 2003; Bhatia et al., 2003; Bidwell et al., 2004; Broadhurst et al., 2004; Fernando et al., 2006a,b, 2010; Xu et al., 2006a,b; Smart et al., 2010). For example, Küpper et al. (2000) described the accumulation of Zn and Cd in leaves of the hyperaccumulator *Arabidopsis halleri* (L.) O'Kane and Al-Shehbaz Novon (Brassicaceae), and found the strongest deposition in trichomes, and to a lesser extent in the mesophyll. Bidwell et al. (2004) found Ni predominantly localized in the leaf epidermal vacuoles with some apoplastic localization in the mesophyll tissues of *Hybanthus floribundus* (Lindley) F. Muell (Violaceae). These and numerous other studies demonstrated that leaf epidermal cell-layers and associated structures such as hairs and trichomes are primary sequestration sites for hyperaccumulated elements, i.e., where highest localized concentrations were detected *in vivo*. Such findings are consistent with reasoning that, (a) dermal tissues generally have a high apoplastic component, (b) the essentially non-photosynthetic status of dermal tissues renders them suited to storing potentially toxic concentrations of metals that might otherwise disrupt vital physiological processes in the mesophyll, and (c) excess metal storage in the dermal layers may contribute to chemical defence against animal and/or insect herbivory. In contrast to all other hyperaccumulators examined to date, Mn hyperaccumulators exhibit a distinct variety of primary detoxification strategies, i.e., in photosynthetic tissues, in non-photosynthetic tissues, and simultaneously across all tissue types (Fernando et al., 2008). Currently there is no clear explanation for this observation. It has been argued that the highly vacuolated xerophytic leaf anatomies of woody Mn hyperaccumulators along with the essential role of Mn in photosynthesis might be associated with its concentration in photosynthetic cells. Most recently however, PIXE/EDS localization studies showed that primary foliar-metal sequestration in a Co-, Ni- and Zn-accumulating Mn hyperaccumulator tree *Gossia fragrantissima* (F. Muell. ex Benth.) N. Snow and Guymer (Myrtaceae) occurred in photosynthetic cells for Mn, Ni and Co; and in non-photosynthetic cells for Zn (Fernando et al., 2013). In further illuminating primary Mn-sequestration in the leaf mesophyll cells of a woody Mn hyperaccumulator, these latest findings indicate involvement of tonoplastal metal transporters not exclusive to Mn since in this species at least, Ni and Co were in addition to Mn, primarily located in photosynthetically important cells.

## Localization studies on Mn hyperaccumulators

As discussed earlier, original *in vivo* examination of foliar Mn microdistribution was performed on Mn accumulators from Japan, including *Chengiopanax sciadophylloides* (Franch. and Sav.) C. B. Shang and J. Y. Huang (Araliaceae) now identified as a “true” Mn hyperaccumulator (Mizuno et al., 2008). However, these samples were prepared by freezing leaf material directly in liquid nitrogen, an approach now regarded as unreliable in achieving adequate cryo fixation at the cellular level (see Echlin, 1992). About 25 years later, *Gossia bidwillii* (Benth.) N. Snow and Guymer (Myrtaceae), a Mn-hyperaccumulating Australian rainforest tree; and *P. acinosa* Roxb, a herbaceous Chinese Mn hyperaccumulator were examined in separate Mn localization studies utilizing cryo-SEM/EDS and synchrotron radiation X-ray fluorescence spectroscopy (SRXRF), respectively, (Fernando et al., 2006b; Xu et al., 2006a,b). Whilst the latter authors showed foliar Mn to be most strongly deposited in the upper epidermis of *P. acinosa*, they also conceded that their sample preparation might have been a confounding factor. Methodology lacking the essential initial step of immediate rapid-freezing of leaf tissue will likely yield unreliable data as a result of artifactual movement of cell solutes and water during subsequent sample processing and/or microbeam analysis. Studies on woody Mn hyperaccumulators utilized appropriate sample fixation that retained cellular content in close representation of the *in vivo* status. Leaf anatomical features of these species typically include substantial cuticles, particularly for the New Caledonian group; multiple layers of large palisade mesophyll cells, and an overall distinct lack of intercellular spaces throughout (Fernando et al., 2006a,b, 2007b, 2008, 2012). These leaves are dermally fortified around closely packed and highly vacuolated cells that account for an overall high vacoular component of the total leaf volume. After field sampling, it is often necessary to maintain material in cool humid packaging over transit periods of least 24 h between remote field collection sites and laboratories where they are immediately cryo-fixed prior to storage or any subsequent processing. Their physical robustness combined with highly localized cellular Mn concentrations that fall well within the detection limits of widely used microprobe methodologies render leaf samples of these woody Mn hyperaccumulators ideal for *in vivo* microprobe localization studies on bulk specimens. Their characteristic leaf sclerophylly on the other hand poses challenges to sample infiltration steps essential to ultrastructural examination by transmission electron microscopy (TEM), and/or analysis by scanning transmission electron microscopy (STEM) EDS.

## Leaf sample preparation for in vivo microprobe examination of hyperaccumulators

Appropriate sample preparation is mandatory to obtaining reliable *in vivo* microprobe analytical data from biological material. Reference to “sample” here should be taken to mean leaf tissues. Sample preparation methodology can determine the degree of anatomical resolution achievable by SEM, as can instrumentation. The latter also governs the limits of analytical resolution and sensitivity, for example, factors such as operating conditions, the choice of incident microbeam, detector and analyzer. There is ample evidence that biological sample preparation for *in vivo* microprobe analysis requires initial rapid cryo-fixation to immobilize metabolic processes and prevent delocalization of diffusible elements (e.g., Echlin et al., 1980; Marshall, 1980a; Echlin, 1992; Marshall and Xu, 1998). Plunging samples directly into liquid nitrogen, while commonly used, does not achieve rapid cryo-fixation since the sample at room temperature upon contact with liquid nitrogen forms a self-enveloping “nitrogen gas vapor cloud,” which has an insulating effect not conducive to rapid freezing. This is known as the “Leidenfrost effect”. Plunge- and metal-mirror contact-freezing methods can be employed to achieve rapid sample vitrification free of ice crystals, which can lead to cell content relocation and membrane rupture. It is desirable to maintain as small a sample size as possible to achieve artifact-free vitrification. Following appropriate cryo-fixation, samples can then be used for analysis after surface polishing or sectioning by cryo-ultramicrotomy. Bulk frozen tissue is cryo-planed to optimize X-ray collection by the detector since a rough sample surface causes irregular absorption, which can then produce inconsistent analytical data. Planing is done cross-sectionally to enable examination of the *in vivo* spatial distribution patterns of elements including Mn through the leaf tissue. Samples can also be processed from a frozen cryo-fixed state to a dry state, depending on the constraints and requirements of an investigation. Whether wet or dry, samples are commonly surface-coated with a fine conductive layer of metal or carbon to avoid charge accumulation from the incident beam provided it does not confound analytical data.

**Figure 1:**
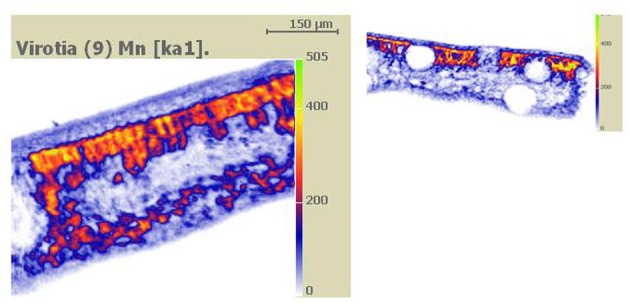
**Dry specimen preparation.** Figure taken from Fernando et al. (2006a). Qualitative PIXE X-ray Mn maps of carbon-coated hand-cut leaf cross-sectional surfaces of cryo-fixed and freeze-dried *Virotia neurophylla* (LHS) and *Gossia bidwillii* (RHS) samples. High Mn deposition is represented as orange to yellow (highest).

**Figure 2:**
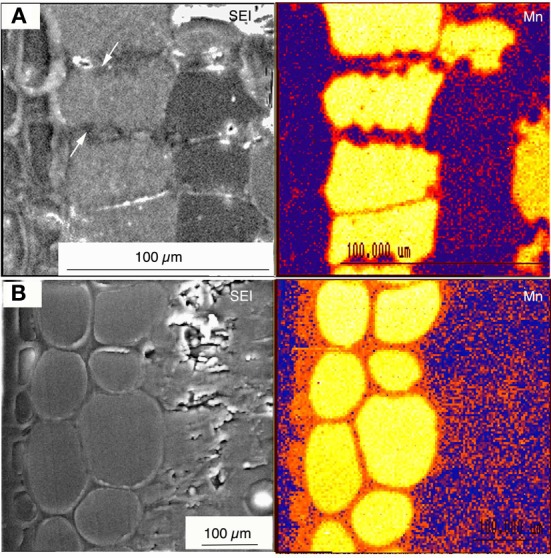
**Wet specimen preparation.** Figure taken from Fernando et al. (2012). Cryo-fixed frozen hydrated and planed leaf cross sectional surfaces of *Virotia neurophylla* **(A)** and *Maytenus fournieri* **(B)** lightly surface sublimed and Al-coated. LHS panels show cryo-SEM images (15 kV); and RHS panels show corresponding quantitative X-ray maps with localized vacuolar Mn highlighted in orange to yellow (highest).

**Figure 3:**
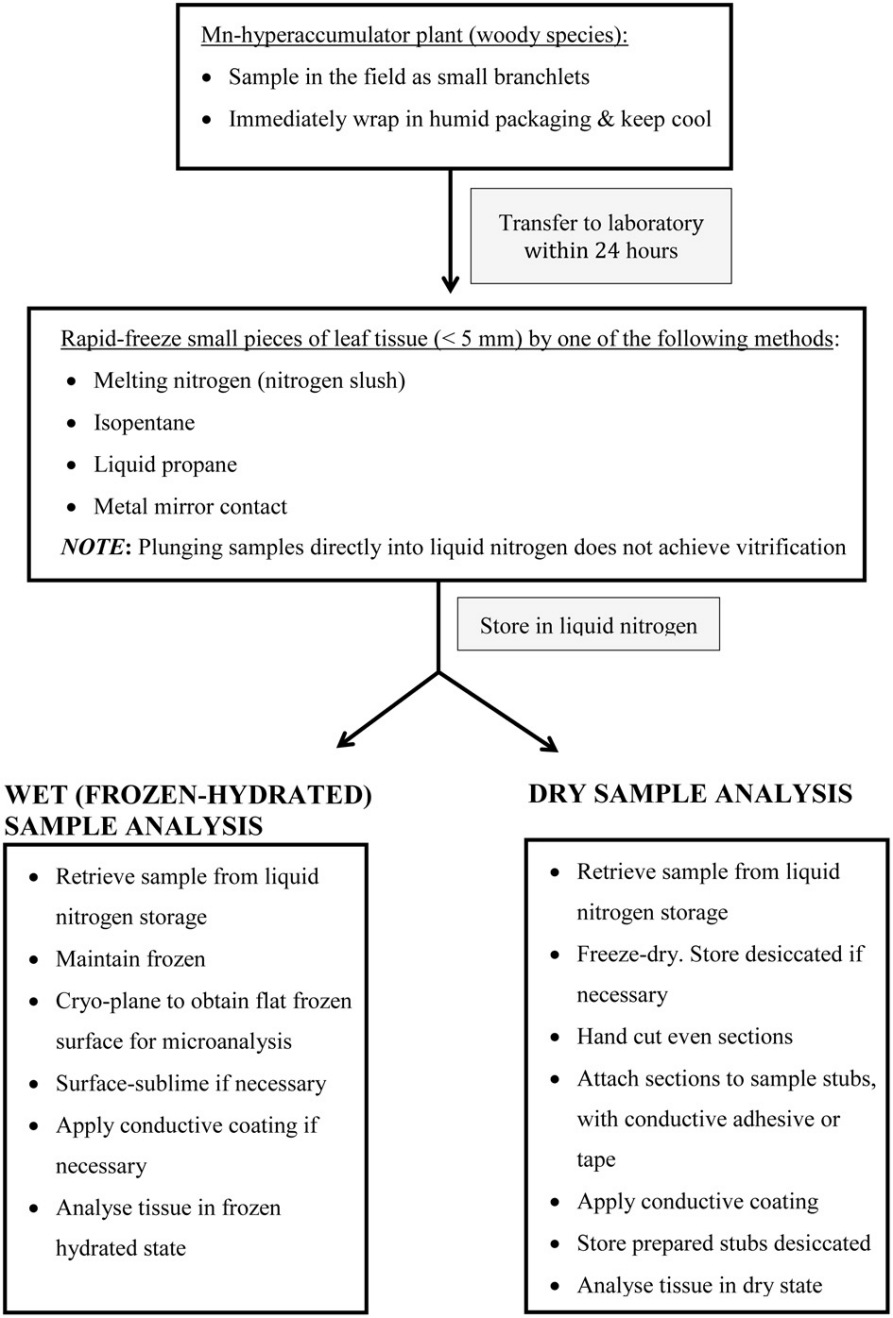
**Schematic summary of important steps in preparing (woody) Mn hyperaccumulator leaf-tissue for *in vivo* microprobe analysis**.

Melting nitrogen, liquid propane and isopentane have previously been used to cryo-fix Mn hyperaccumulator samples for wet and dry sample preparations examined by qualitative and quantitative electron and proton probe studies (Fernando et al., 2006a,b, 2013). Whether applied to wet or dry samples, quantitative approaches are more time consuming and expensive. Qualitative X-ray mapping used on freeze-dried bulk tissue cut open to expose a clean cross-sectional surface has to date been best exploited to locate primary Mn sinks in Mn hyperaccumulator samples (Figure 1) (Fernando et al., 2006a, 2007b, 2008). Again, it is noteworthy that the sclerophyllous nature of these leaves is a distinct advantage when hand-cutting freeze dried tissue. This is a relatively straightforward yet effective approach given that Mn hyperaccumulators harbour highly localized foliar Mn. If necessary, these findings can then be followed up by quantitative techniques to characterize Mn sinks in greater detail, using additional samples prepared appropriately. For example, SEM/EDS measurement of vacuolar elemental concentrations within individual cells can be carried out on frozen hydrated cryo-planed samples treated to improve anatomical clarity by superficial surface-sublimation and metal coating (Figure 2) (Fernando et al., 2006b, 2012, 2013). A preparation such as this offers minimal disruption to metabolic processes, and is the closest achievable representation of the true *in vivo* state for a fixed sample, provided it was correctly cryo-fixed at the outset. Among the disadvantages of this approach however, is that the sample water content has a “diluting” effect that renders cellular elements less detectable than in correspondingly dry tissue, although Mn is generally localized in sufficiently high concentrations to be easily detected even in fully hydrated tissue. Here, Figures 1, 2 are presented side-by-side to reinforce the point that leaves of woody Mn hyperaccumulators by virtue of their physical robustness, leaf anatomy combined with extraordinary foliar Mn concentrations, are almost uniquely equally well suited to both wet and dry sample preparation for *in vivo* analyses—be they qualitative or quantitative. Hydrated samples are advantageous in that the analytical resolution is higher than in freeze-dried samples since it is partially determined by sample depth. However, high incident beam energies can lead to beam damage on the sample (Marshall, 1980c). Küpper et al. (1999, 2000, 2001) and Robinson et al. (2003) have similarly employed cryo-SEM/EDS on cryo-fixed frozen samples to examine Ni, Cd, and Zn localization in herbaceous hyperaccumulators, and achieved subcellular spatial resolution of metal distribution in hydrated tissues. Freeze drying cryo-fixed material on the other hand, while elevating analytical detection limits, leads to further loss of analytical resolution when the water matrix is removed from cells. Analysis of sections using STEM enables examination of tissue ultrastructure with cell contents intact. This is achieved by anhydrous freeze-substitution as used by Bidwell et al. (2004) to quantify foliar Ni sequestration *in vivo* in a herbaceous Ni hyperaccumulator. In an initial process of cryo-substitution, tissue-water as ice is slowly replaced with a non-polar solvent, subsequently followed by resin infiltration (Pallaghy, 1973; Marshall, 1980b). Thick sections (1–2 μm) can then be used for energy dispersive analysis in STEM. It is unlike TEM sample preparations routinely used for anatomical/ultrastructural studies, in which cellular ions are washed out in polar solvents such as acetone and ethanol (Morgan, 1980). The authors' previous experience in attempting to infiltrate sclerophyllous leaf tissues of woody Mn hyperaccumulators for TEM studies were unsuccessful due to the apparent impermeabilty of samples. Major considerations when preparing woody hyperaccumulator plant material for *in vivo* microprobe analysis is broadly summarized in Figure 3, however, caution needs to be exercized when interpreting these steps for other material and species. For example, “soft” herbaceous plant material while more permeable to fixatives, requires far more rapid handling between collection sites and laboratory, and are considerably less suited to dry hand-sectioning.

## Mn transporter studies and future directions

The accumulation of shoot tissue Mn concentrations significantly above normal plant nutritional requirements is not an uncommon observation that raises questions about transporters associated with foliar Mn compartmentalization and specificity at the root-soil interface. Plants that hyperaccumulate Mn can be viewed as exceptional in this regard. There has been little success in the search for Mn transporters responsible for Mn acquisition by model plants. As reviewed by Pittman (2005), certain classes of divalent-metal transporters such as the ZIP (ZRT1/IRT1-related protein) and NRAMP (natural resistance-associated macrophage protein) appear to be implicated in Mn transport. Recently, a group of NRAMP transporters including OsNramp5 in rice (Ishimaru et al., 2012; Sasaki et al., 2012), and AtNramp1 in *Arabidopsis thaliana* (Cailliatte et al., 2010) were found to be involved in Mn uptake at the root surface. The NRAMP group is recognized as being associated with heavy metal transport and possibly contributing to plant adaptation to metal homeostasis (Thomine et al., 2000). In certain instances they are more highly expressed in metal-hyperaccumulating plants than in models such as *A. thaliana* (Weber et al., 2004; Oomen et al., 2009). This suggests that NRAMP transporters are important to metal hyperaccumulation either at the uptake level, root to shoot translocation, or detoxification via vacuolar compartmentation. Similarly, the ZIP transporters are known to be highly expressed in metal hyperaccumulators. However, since neither NRAMP nor ZIP transporters appear to be highly expressed in Mn hyperaccumulators, there is little evidence of their involvement in Mn-hyperaccumulative uptake and/or accumulation (T. Mizuno, pers. comm.). Delhaize et al. (2003) identified ShMTP8 as a vacuolar transporter responsible for Mn-accumulative tolerance in *Stylosanthes hamata* (Fabaceae), and also described AtMTP11 as a Mn transporter in prevacuolar compartments of *Arabidopsis* (2007). Peiter et al. (2007) found that the Mn transporter MTP11 was associated with Golgi bodies in *Arabidopsis mtp 11* mutants exhibiting an enhanced ability to accumulate Mn in their shoots and roots.

*In vivo* microprobe localization methodologies have expanded knowledge about the compartmentalization of excess foliar Mn in hyperaccumulators by revealing remarkable tonoplastal specificity within certain cell types, particularly the palisade mesophyll (Fernando et al., 2006a,b, 2012). These findings provide basis for future genetic studies to identify drivers of Mn hyperaccumulation, which could ultimately contribute to discussion on plant Mn accumulation in the context of Mn crop toxicity. Discovery that foliar Mn is variously co-localized with excess Zn, Co, and Ni in different palisade cell-layers of a Mn hyperaccumulator provides further insight into metal transporters (Fernando et al., 2013). Currently there is no published research on the genetic basis of Mn hyperaccumulation. With a single exception, Mn transporter studies in yeast, plant models (Delhaize et al., 2003, 2007; Mizuno et al., 2005; Pittman, 2005), and the Mn hyperaccumulator *C. sciadophylloides* (T. Mizuno, pers. comm.) have largely been unsuccessful in characterizing the molecular basis of detoxification mechanisms associated with excess foliar Mn sequestration in Mn-accumulating plants.

### Conflict of interest statement

The authors declare that the research was conducted in the absence of any commercial or financial relationships that could be construed as a potential conflict of interest.
